# Antibodies against *Marinobacter algicola* and *Salmonella typhimurium* Flagellins Do Not Cross-Neutralize TLR5 Activation

**DOI:** 10.1371/journal.pone.0048466

**Published:** 2012-11-14

**Authors:** Raul Terron-Exposito, Benoit Dudognon, Inmaculada Galindo, Jose I. Quetglas, Julio M. Coll, Jose M. Escribano, Eduardo Gomez-Casado

**Affiliations:** 1 Department of Biotechnology, Instituto Nacional de Investigación y Tecnología Agraria y Alimentaria, INIA, Madrid, Spain; 2 Alternative Gene Expression S. L. (ALGENEX S. L.), Madrid, Spain; 3 Division of Gene Therapy, Centro de Investigación en Medicina Aplicada, CIMA, Pamplona, Spain; Indian Institute of Science, India

## Abstract

Flagellins evoke strong innate and adaptive immune responses. These proteins may play a key role as radioprotectors, exert antitumoral activity in certain types of tumor and reduce graft-versus-host disease in allogeneic hematopoietic stem cell transplant recipients. Notwithstanding, flagellins are highly immunogenic, and repeated use leads to their neutralization by systemic antibodies. This neutralization is not prevented by using functional deleted flagellins. These observations led us to explore the possibility of preventing initial neutralization by means of another functional flagellin that does not belong to common pathogenic bacteria but that has the capacity to activate TLR5. Here we characterized the functional capacity of the two-phase *Marinobacter algicola* (MA)-derived flagellins (F and FR) as systemic and mucosal adjuvants and compared their performance with that of *Salmonella typhimurium* (STF) flagellins (FljB and FliC). We also report for the first time on the *in vitro* and *in vivo* capacity of various flagellins to trigger TLR5 activation in the presence of species-specific anti-flagellin antibodies, the cross-neutralization mediated by these antibodies, and the sequential use of these flagellins for TLR5 activation. Our results showed that MA flagellins behave in a similar way to STF ones, inducing pro-inflammatory cytokines (IL8, CCL20, CCL2) and evoking a strong *in vivo* antibody response against a model epitope. More importantly, MA flagellins were fully functional, *in vitro* or *in vivo*, in the presence of a high concentration of neutralizing anti-flagellin STF antibodies, and STF flagellin was not inhibited by neutralizing anti-flagellin MA antibodies. The use of active flagellins from distinct bacteria could be a useful approach to prevent systemic neutralization of this group of adjuvants and to facilitate the rational design of flagellin-based vaccines and/or other therapeutic treatments (against ischemia, acute renal failure, tumors, ionizing radiations and also to improve the outcome of bone marrow transplants).

## Introduction

Flagellin proteins are the major structural component of the flagellum, an appendage that confers motility to bacteria [1]. Some flagellins have the capacity to activate the Toll-like receptor 5 (TLR5), which is present at the surface of epithelial, osteoblasts, antigen-presenting cells (APC), and immune cells [2]–[4]. Flagellin-induced TLR5 activation leads to signal transduction pathways that involve NF-κβ, which finally trigger the expression of cytokines and co-stimulatory molecules [5], [6], thus increasing both humoral and cell-mediated immune responses [7]–[10]. Flagellin plays a key role in radioprotection as it suppresses the apoptosis that takes place as a result of high-dose ionizing radiation in acute radiation syndromes, such as those that occur in the hematopoietic and gastrointestinal cells [11]–[13]. Flagellin improves reperfusion, thus decreasing ischemia and acute renal failure [14], [15]. Moreover, this protein reduces graft-versus-host disease (GVHD) in recipients of allogeneic hematopoietic stem cell transplants while enhancing antiviral immunity against a mouse cytomegalovirus (CMV) model [16]. Some studies have attributed broad antimicrobial effects [12] and antitumoral activity [17]–[20] to flagellin while others have reported pro-tumoral activity [21].

Flagellin has been used as a broad adjuvant against multiple pathogens like *Yersinia pestis* [22], West Nile virus [23], influenza [10], [24]–[26], malaria [27], [28], *E. coli* [29] and also against aflagellated bacterium such as *Streptococcus pneumoniae* [30]. Since flagellins are also considered to be virulence factors [31], other flagellin vaccines have been used against pathogenic flagellated bacteria that affect mammals, such as *E. coli* [29], *Salmonella* sp [32], *P. aeruginosa* [33]–[35], *Vibrio vulnificus* [36], *Burkholderia pseudomallei* [37], and also sea animals, such as *Edwardsiella tarda* [38] and *Vibrio alginolyticus* [39], [40]. Flagellins belonging mainly to alpha- and epsilon-proteobacteria (i.e. *H. pylori, H. mustelae, and C. jejuni*) evade TLR5 recognition because they carry some changes in their primary amino acid sequence at domain D1 (between amino acids 89 and 96) that make them unable to bind to TLR5; however, they preserve their motility and can spread without inducing an innate immune response [4]. To date, most flagellin-based vaccines or flagellin-TLR5 activators that have been addressed deal with *Salmonella typhimurium* flagellin (STF) C (FliC). The wide use of FliC is attributable to the fact that it was one of the first flagellin models studied [41] and it induces more TNF-α than flagellin proteins from *E. coli*, *P. aeruginosa*, and *Y. enterocolitica* [42].

Unfortunately, flagellin also acts as a potent immunogen, inducing anti-flagellin antibodies that neutralize its adjuvant capacity. Flagellins without their hypervariable region are less immunogenic, but this feature does not prevent neutralization [43]. Moreover, the hypervariable region of flagellins could be crucial in systemic activation [43], [44] and may show different pro-inflammatory effects in mucosa [43]. Flagellin neutralization implies that the exposure of an individual to the other common flagellated bacteria named above, as a result of infection and reinfection or by vaccination, will also give rise to the neutralization of their flagellin molecules.

Vaccination through the intra-nasal (i.n.) route seems to be the best approach to stimulate mucosal immunity because this route circumvents flagellin neutralization by serum antibodies, and IgA antibodies are unable to inhibit the flagellin function [43], [45]. However, commensal bacteria and their flagellin proteins have recently been shown to be well tolerated in the gut, possibly as a result of a population of regulatory flagellin-specific T-lymphocytes capable of increasing soluble IgA (sIgA), which inhibits continuous inflammation [46]. Furthermore, the administration of flagellin by the systemic route allows the treatment of tumors [18], [19] and rapid delivery of a high concentration to activate NF-κβ, [11], which is required to inhibit the apoptosis caused by a high-dose of ionizing radiation [11], [13].

The repeated administration of flagellins induces antibodies, which neutralize the function of these proteins. Thus research into alternative flagellin molecules is pertinent. One possible strategy is to use two flagellin proteins from different bacteria when the antibodies raised against one of them do not neutralize the other. Therefore, the use of flagellins from distinct bacteria could contribute to a more rational design of flagellin-based vaccines and/or other therapeutic treatments (e.g. antitumoral therapies and treatments against ionizing radiations). However, whether hyperimmunization to flagellin of one bacterial species neutralizes the effect of a flagellin protein from a distinct bacterial species remains unknown. In this regard, here we studied the functionality and behavior of the flagellins from *Marinobacter algicola* (MA), a non-pathogenic bacteria isolated from the marine medium that can be grown in safe conditions. Described in 1992, MA is a gram-negative, aerobic, halophilic gamma proteobacterium capable of degrading a variety of hydrocarbons [47]. MA was first isolated from the bacterial flora associated with the dinoflagellate *Gymnodinium catenatum* Graham [48], recovered from the Yellow Sea, Korea. More than 21 MA species have been described to date [49].

Here we characterized the functional capacity of the two-phase flagellins (F and FR) from MA as systemic and mucosal adjuvants and compared their performance with that of STF flagellins (FljB and FliC). Thus, we have also studied for the first time the *in vitro* and *in vivo* capacity of flagellins of diverse origin to activate TLR5 in the presence of homologous and heterologous anti-flagellin antibodies.

Our results demonstrated that MA flagellins have a similar capacity to STF ones regarding TLR5 activation, as shown by the induction of similar levels of cytokine expression (IL8, CCL2, CCL20). They also induce a similar level of IgG antibodies against a model immunogen. More importantly, neither MA flagellins are neutralized by anti-STF flagellin antibodies nor STF flagellin is neutralized by anti-MA flagellin antibodies. The use of active flagellins from these bacteria (MA, STF), independently or sequentially (prime-boosts), could be useful for the rational design of flagellin-based vaccines and other therapeutic treatments (antitumoral, against ionizing radiations, for improving transplants and reperfusion), thereby circumventing systemic neutralization of the adjuvant.

## Materials and Methods

### Ethics statement

All animal experiments were approved by the ethical and biosecurity committee from INIA and were performed following the guidelines of the European Commission (directives 86/609/ECC and 93/119/ECC). Mice were maintained under pathogen-free conditions and allowed to acclimatize to the biosafety level 2 (BSL2) animal facilities at the Department of Animal Reproduction from INIA (accreditation number 28079-36-A) for 1 week before use in our experiments. Sacrifices were carried out using CO_2_ inhalation at concentration above 70%, and all efforts were made to minimize suffering.

### Production of recombinant flagellins

MA (F and FR) and *Vibrio vulnificus* (Vvul) flagellin genes were chemically synthesized (MrGene, Germany) from previously described primary sequences (accession numbers NZ_ABCP01000018 and NC_005139 DNA, respectively). In order to clone the nucleotide sequences into pFastBac™1 plasmid (Invitrogen, USA), they were synthesized with restriction enzymes 5′-Bam HI/3′-Hind III. Flagellin (FljB) from the *Salmonella enterica* serovar *typhimurium* (STF) was obtained from genomic DNA by PCR using the following primer pairs: STF2BamHI (5′GCCGGATCCATGGCACAAGTAATCAACAC), and 3STF2Ecorc (5′GCGGAATTCACGTAACAGAGACAGCAC). PCR conditions comprise an initial denaturation step of 5 min at 96°C followed by 30 cycles of 96°C for 20 s, 60°C for 30 s, and 72°C for 45 s with a final extension step of 10 min at 72°C.

Recombinant pFastbac™1 plasmids were used to generate the recombinant baculoviruses by means of the Bac-to-Bac® Baculovirus system (Invitrogen, USA), following the manufacturer's instructions. Recombinant baculoviruses were propagated and amplified in Sf21 insect cells to reach infective titers of 10^8^ pfu/ml and stocks were kept at 4°C for daily use and at −80°C for long-term storage. In order to express the recombinant proteins, Sf21 cells were infected at a multiplicity of infection (MOI) of five and harvested 72 h post-infection.

The FljB_Δ180–400_ and FljB_Δ220–320_ deletion mutant flagellins were obtained by PCR amplification from a previously amplified STF (FljB) amplicon. FljB_Δ180–400_ was obtained using the specific primers STF2BamHI, R180EcoRI (5′GTCGAATTCAGCTTTCGTTGTTACTGCT), F400EcoRI (5′GACGAATTCGATTTCAAAGCACAACCAG), and R320XbaIHindIII (5′CTAAAGCTTCTATCTAGAACGTAACAGAGACAGCAC). Otherwise, FljB_Δ220–320_ deletion mutant flagellin was obtained using STF2BamHI, R220EcoRI (5′GTCGAATTCGGTTACAGAAGCCGTACCA), F320EcoRI (5′GACGAATTCTATACCGATAAAAATGGTAAGACA) and R320XbaIHindIII.

These flagellin fragments were cloned into pRSET-A vector (Invitrogen, USA). Recombinant clones were transfected into BL21 (DE3)pLysS cells and grown overnight at 37°C in Luria–Bertani medium with appropriate antibiotics. Logarithmic-phase growing bacteria were induced with 1 mM isopropyl β-D-thiogalactoside (IPTG).

All sequences held a 6His-tag followed by the amino acid sequence KDEL (lys-Asp-Glu-Leu) at their 3′-end [50], [51]. Retention of this sequence in the endoplasmic reticulum may prevent degradation and therefore increase the amount of protein [50], [51]. All fusion proteins were prepared and purified by affinity chromatography on a Co^2+^ resin (HisPur cobalt resin, Pierce, USA). Quantification of the purified fusion proteins was completed by the Bradford method [52] and Coomassie blue staining by comparison with a BSA standard curve.

All the flagellins used for *in vivo* experiments were produced in baculovirus-insect cell system instead of bacteria to avoid bacterial LPS contamination. The presence of LPS may produce *in vivo* an undesirable immune response. In addition, higher yields and purity of the flagellins were obtained from the recombinant baculoviruses. Flagellins produced in *E. coli* (FljB_Δ220–320_, FljB_Δ180–400_) were only used *in vitro* as antigens for comparison with FljB in ELISA.

Phase-2 flagellin (FliC) from STF was purchased from Alexis Biochemicals (Enzo Life Sciences International, Inc., USA). Contaminating lipopolysaccharides (LPS) were removed from the recombinant proteins using the Affinity Pak Detoxi-Gel Endotoxin Removing gel (Pierce Biotechnology, Inc., Rockford, IL), and the residual LPS content of the protein was determined by the ToxinSensor™ Chromogenic LAL Endotoxin Assay Kit (GenScript, NJ, USA). The LPS levels in flagellin preparations were <0.2 EU/ml.

### Immunization and hyperimmunization

Female BALB/C mice (6–8 weeks old) were purchased from Harlan Laboratories (Barcelona, Spain). To study the capacity of MA flagellins to induce antigen-specific antibodies, we generated fusion flagellins with four copies of the DUD (4DUD) peptide (Dynein Union Domain, HPTEPYTTVTTQNTASQTMSAIENL) as a model (the underlined sequence is responsible for binding to dynein). This DUD motif is present in the p54 protein of African swine fever virus and is a very poor immunogenic peptide in natural and experimental infections [53], [54]. Use of four copies of a peptide increases the immunogenicity when compared to only one copy [55].

For the immunization assays, mice (5 per group) were injected subcutaneously (s.c.) with 10 µg/100 µl PBS of the fusion flagellins of STF FljB4DUD, and MA F4DUD and FR4DUD on days 1, and 15. A control group was inoculated s.c. with the equivalent moles of 4DUD in 100 µl PBS.

For flagellin hyperimmunization, animals (5 per group) were injected s.c. (10 µg/injection) with the flagellins FljB (STF), F and FR (MA) emulsified in 100 µL of incomplete Freund's adjuvant (IFA)/PBS on days 1, 21, 35, 49 and 63. Antibody expression in each mouse was evaluated by ELISA against various flagellins at distinct times after immunization. These immunizations were repeated to reach antibody (IgG) titers between 1/50,000 and 1/100,000.

To assess the innate immune response, we measured the early expression of the CCL20 cytokine induced by flagellins in flagellin-treated hyperimmunized mice. This chemokine recruits immature dendritic cells and lymphocytes to target sites [43]. Serial dilutions of hyperimmune serum (in 100 µl of PBS) were passively transferred by injection to naive mice (5 per group) by the retro-orbital route. After 1 h, mice were inoculated with 50 ng of FljB or FR by the same route. We studied 3 groups of animals: 1) those passively transferred with anti-STF FljB sera and inoculated with MA FR; 2) those passively transferred with anti-MA FR sera and inoculated with STF FljB; and 3) those passively transferred with anti-STF FljB sera and inoculated with STF FljB. After 6 h of the inoculation of the flagellin, the mice were bled to collect sera. All serum samples were decomplemented at 56°C and further stored at −80°C before analysis.

In addition, to assess the capacity of the flagellin molecules to activate TLR5 *in vivo*, FljB- hyperimmune mice (5 per group) were injected s.c. with 4.8 µg of FR, FljB or Vvul flagellins, and CCL20 expression was measured in sera after 6 h.

Analysis of Ag-specific Ab responsesAntibody expression against FljB, F, FR, and the model DUD peptide were assessed using ELISAs. Briefly, 200 ng/well of p54 purified protein (containing the DUD sequence) or the flagellins (FljB, FljB_Δ180–400_, FljB_Δ220–320_, FliC, F and FR) were coated on Maxi-Sorp microplates (Nalgene, Nunc, Denmark) overnight at 4°C in carbonate buffer. All microplates were washed with PBS/0.05% Tween 20 (PBST) and then blocked with PBST/2% BSA (blocking solution) for 1 h at 37°C under rotation. Serial dilutions of serum samples at 100 µl per well were incubated for 1 h at room temperature and then washed with PBST. HRP-conjugated antimouse IgG antibody (GE Healthcare, USA), diluted 1∶2000 in blocking solution, was finally added. Plates were washed four times with PBST, and 100 µl/well of 1 mM 2,2′-Azino-bis (3-ethylbenzothiazoline-6-sulfonic acid) (ABTS, KPL, USA) was added to the plates. Peroxidase reaction was allowed to develop for 15 min at room temperature and was then read at 405 nm (OD_405_) in an ELISA microplate reader (Multiskan EX, Thermo Electron Corp, USA). The cut-off discriminating negative and positive sera were set at 2–3 times the mean of the OD_405_ value from sera from pre-immune or DUD peptide immunized mice (controls).

### Cell-based assays

The Caco-2 human colon adenocarcinoma cell line (a gift of Dr. J.M. Martin-Villa, Medicine, UCM, Madrid. ATCC number HTB-37™) was used for TLR5 stimulation and inhibition assays with MA and STF flagellins. These intestinal epithelial cells were grown in DMEM supplemented with 5% FCS, 10 mM HEPES, 1× non-essential amino acids, 100 U/ml penicillin, and 100 U/ml streptomycin. Cells (2×10^5^/well) were incubated with the recombinant flagellins (FljB, FR and F) at distinct concentrations (1000, 333, 166, and 100 ng/ml) for 16 h before harvesting the supernatants for cytokine measurement by ELISA.

### Cross neutralization assays with the anti-flagellin-specific antibodies

For cross-neutralization assays, flagellins (FljB, F and FR) at different concentrations (500, 166, 83 and 50 ng) were mixed and incubated at 37°C for 1 h with heat-inactivated hyperimmune sera (3 µl) in the following combinations: each flagellin (FljB, F or FR) was mixed with anti-FljB antisera; the same flagellins were mixed with anti-FR antisera; and the same flagellins were mixed with anti-F antisera. After incubation, mixtures were used to stimulate Caco-2 cells in the conditions described above.

### Cytokine-specific ELISAs

Human IL8 (CXCL8) and CCL2 (MCP-1), and mouse CCL20 levels were measured in Caco-2 cell supernatants and mouse sera, respectively, using commercial ELISA kits (eBioscence and R&D Systems, respectively). Assays were done following manufacturer's instructions.

### Mouse-IgG quantification

IgG (all subclasses) concentrations in sera collected from hyperimmunized mice were measured using a sandwich ELISA and following the manufacturer's instructions (Roche, Germany).

### Statistical analysis

Statistical differences were analyzed using the one-way ANOVA and the Mann-Whitney *U* tests, and were considered to be significant at p≤0.05. Unless otherwise stated, results are expressed as arithmetic means ± standard deviations (SD). All experiments were repeated two or three times as independent assays.

## Results

### Homology of protein sequences and activating TLR5 domain in MA- and STF-derived flagellins

**Figure 1:**
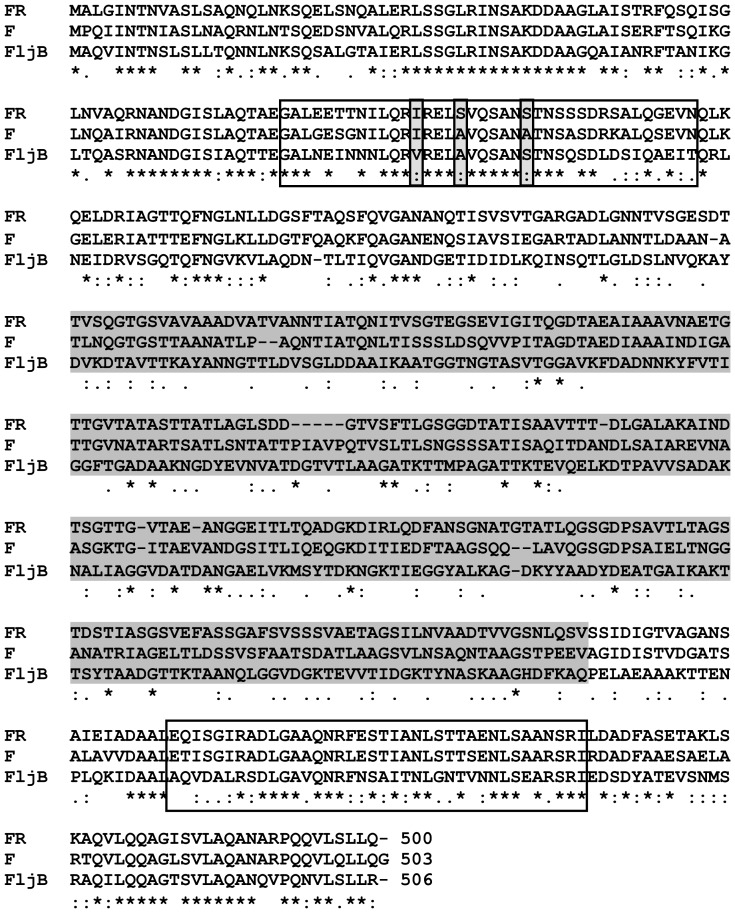
Comparison of the flagellin amino acid sequences of MA (FR and F) and STF (FljB). Boxed areas indicate putative TLR5 binding domains. Grey sequences show hypervariable domains. *,denotes identical amino acids. :, denotes related amino acids.

MA flagellins F and FR comprise 503 and 500 amino acids, respectively. Full-length protein sequences (**Fig. 1**) showed 63% of homology between F and FR, and 34% and 35% between FR and FljB, and between F and FljB, respectively. When conserved sequences (the first 170 amino acids and the last 90 amino acids) were considered, the homology between F and FR was about 76% at the N-terminal and 80% at the C-terminal constant regions. The values for these two terminal regions were 55% and 57% between F and FljB and between FR and FljB, respectively. In contrast, FliC and FljB flagellins shared constant N- and C-terminal regions whereas their hypervariable domains showed many differences. Regarding MA flagellins, hypervariable regions from F and FR shared a low sequence homology (**Fig. 1**).

In addition, with respect to STF, both MA flagellins showed some differences in the D1 domain (amino acids 89–96) of interaction with TLR5 [56]. However, changes at these positions seem to be permissive to the flagellin structure [4] since they did not affect TLR5 recognition (amino acids 92, 96 and 102, see **Fig. 1**, boxed area).

### MA flagellins stimulate IL8 and CCL2 production in epithelial cells

**Figure 2:**
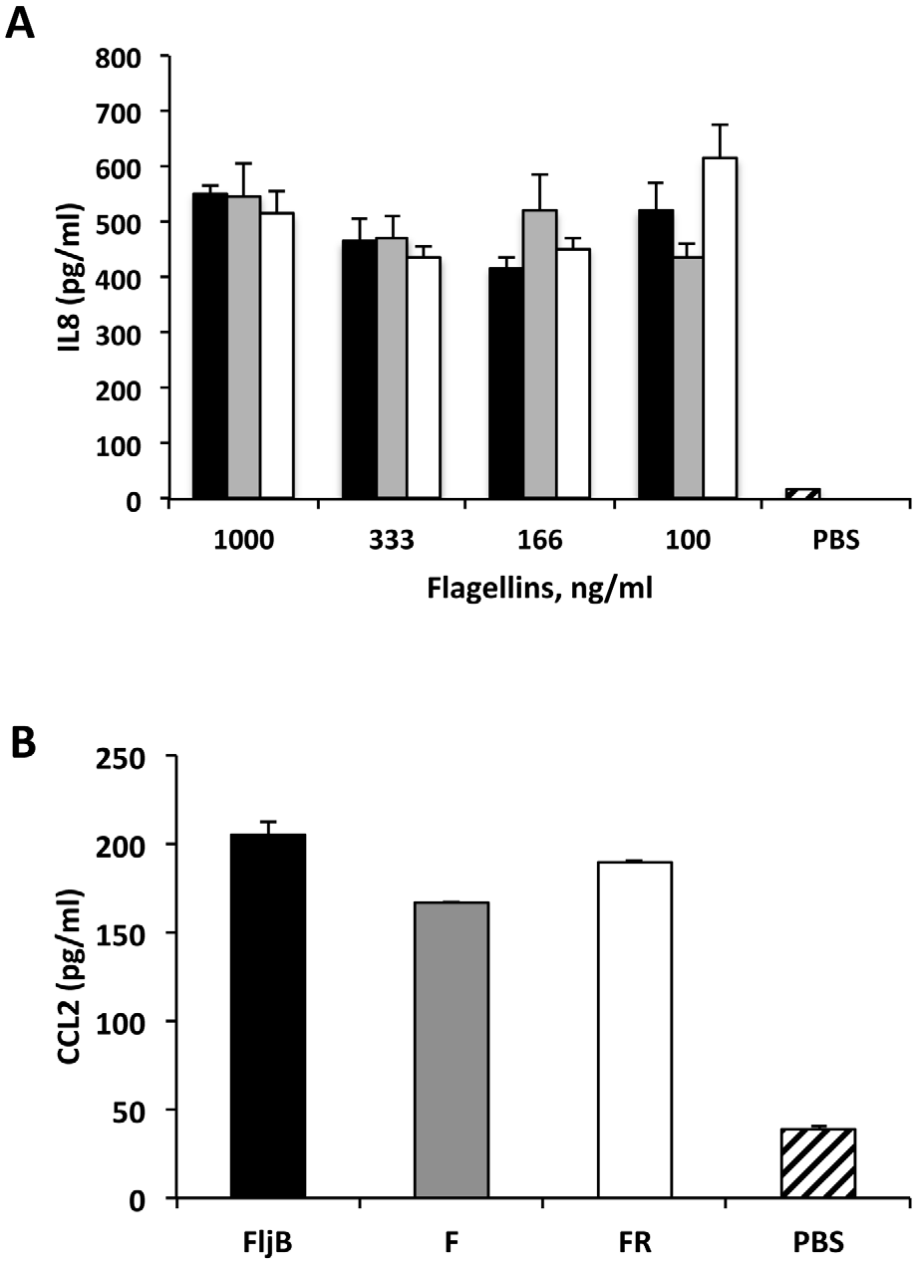
*In vitro* cytokine production in Caco-2 cells stimulated by MA (F, FR) and STF (FljB) flagellins. A) Human IL8 secretion by Caco-2 cells stimulated with FljB (black bars), F (grey bars) and FR (white bars) flagellins. B) Human CCL2 secretion by Caco-2 cells stimulated with FljB, F and FR at 1000 ng/ml. IL8 and CCL2 levels are shown in pg/ml. Differences in cytokine expression were not statistically significant at the p≤0.05 level. PBS, phosphate-buffered saline.

We determined the capacity of MA flagellins to stimulate human Caco-2 cells to produce IL8 and CCL2 cytokines through TLR5 activation [57]. The stimulatory capacity of these proteins was compared to that obtained with STF (FljB) flagellin. Caco-2 cells were treated with a range of flagellin concentrations (from 1000 ng/ml to 100 ng/ml). After stimulation, secreted IL8 and CCL2 in the culture supernatants were determined by ELISA. F and FR induced a similar level of IL8 as FljB at the concentrations tested (**Fig. 2A**). MA and STF flagellins also gave similar results with regards to secreted CCL2 (**Fig. 2B**). Since IL8 and CCL2 are induced after TLR5 stimulation [57], these findings suggest that F and FR activate TLR5 in a similar way to FljB.

### MA flagellins have adjuvant activity both in systemic and mucosal immune compartments

Two fusion proteins with MA flagellins (F4DUD, FR4DUD) were used as vaccination immunogen to study their adjuvant capacity for IgG production against the DUD sequence (see Methods). As a control, we used an STF fusion flagellin with the same model peptide (FljB4DUD).

**Figure 3:**
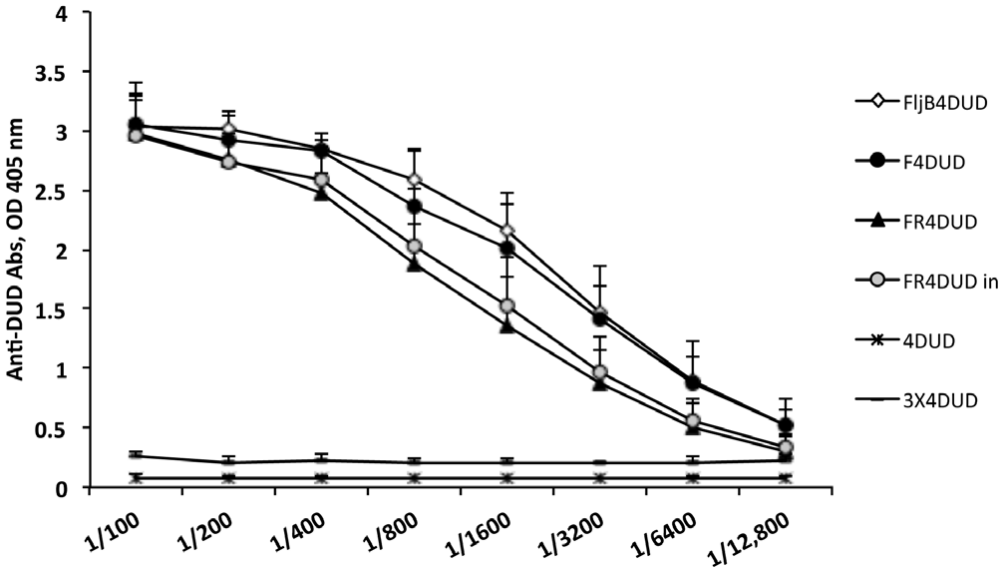
IgG anti-DUD antibodies induced by MA and STF fusion flagellins. Titration of antibody levels against the Dynein Union Domain (DUD) peptide induced in mice after injection with MA and STF 4DUD-fusion flagellins (F4DUD, FR4DUD, and FljB4DUD). ◊, FljB4DUD. •, F4DUD. ▴, FR4DUD. ???, FR4DUDin. *, 4DUD. –, 3×4DUD (3 times 4DUD OD values). Differences of OD values among flagellins were not statistically significant at the p≤0.05 level.

Sera from immunized mice harvested 45 days after two inoculations with either the recombinant fusion flagellins MA (F4DUD) or STF (FljB4DUD) showed similar IgG serum levels and titration profiles when delivered subcutaneously (**Fig. 3**). FR4DUD inoculated by the same route induced a lower titer of anti-DUD antibodies than F4DUD and FljB4DUD. However, in mice immunized by the intranasal (i.n.) route, slightly lower antibody immune responses were observed in comparison with those vaccinated (s.c.) twice with F4DUD (**Fig. 3**). In contrast to tetrapeptide DUD (4DUD), both the MA and STF flagellins fused to the 4DUD peptide induced IgG antibody titers between 1/6400 and 1/12,800. Differences in anti-DUD IgG levels induced by the distinct flagellins were not statistically significant.

### MA- F and STF flagellins show low cross-reactivity

**Figure 4:**
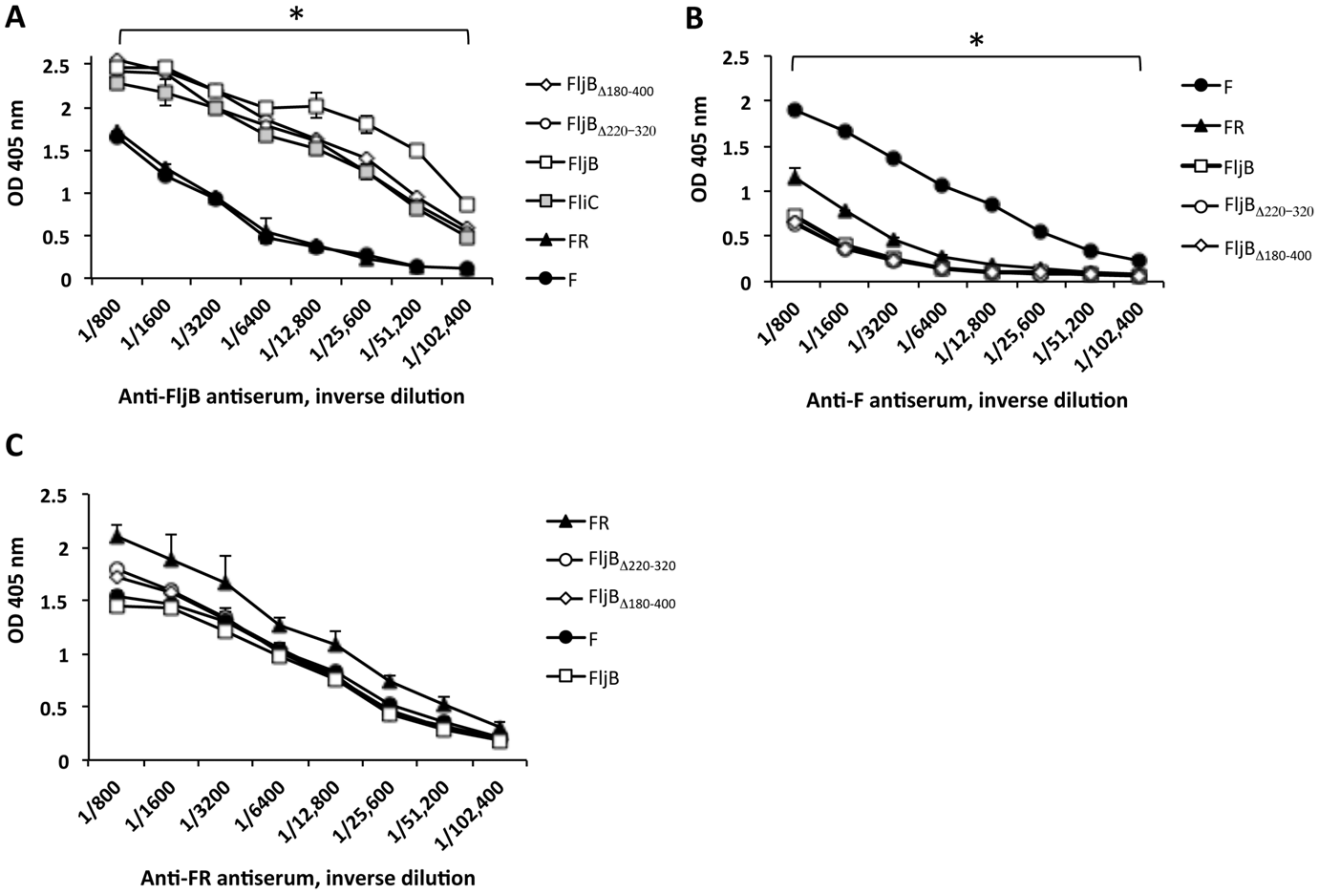
Cross-reaction of the anti-MA and –STF flagellin antisera with these flagellins. Cross-reactivity of anti-FljB (A), anti-F (B) and anti-FR (C) antiserum with MA (F, FR) and STF (FljB, FliC) flagellins (200 ng/well). IgG antibody binding was measured by ELISA. A) *, statistical significance differences, at the p≤0.05 level, between all FljB/FliC STF flagellins and F and FR MA flagellins at each dilution point. B) *, statistical significance differences, at the p≤0.05 level, between F and other flagellins at each dilution point. ◊,FljB_Δ180–400_. ○, FljB_Δ220–320_. □,FljB. ???, FliC. ▴, FR. •, F.

Anti-FljB mouse antibodies bound to FljB in an ELISA test in a similar way to the deleted forms FljB_Δ180–400_, FljB_Δ220–320_ and to FliC. This observation could be explained by the fact that these flagellins share the conserved amino- and carboxy-terminal domains (**Fig. 4A**). Thus, the serum against FljB also contains antibodies with the capacity to bind to the hypervariable region of FljB, thus giving rise to an increased signal with respect to the deleted FljB forms, even at high sera dilutions (**Fig. 4A**). Of note, the anti-FljB antibodies bound to F and FR to a much lower extent than to FljB when using the same amount of flagellin protein for ELISA detection (**Fig. 4A**), being statistically significant this difference of cross-reactivity. This finding suggests that FR and F would be functional, not neutralized, in the presence of neutralizing anti-FljB antibodies.

F flagellin induced IgG antibodies in mouse with an statistical significance degree of recognition of FR or FljB around 8 or 16 times lower, respectively, than F (**Fig. 4B**). In contrast, anti-FR antibodies showed a similar binding capacity for all the flagellins tested (**Fig. 4C**).

### Anti-STF flagellin antibodies do not neutralize MA flagellins

**Figure 5:**
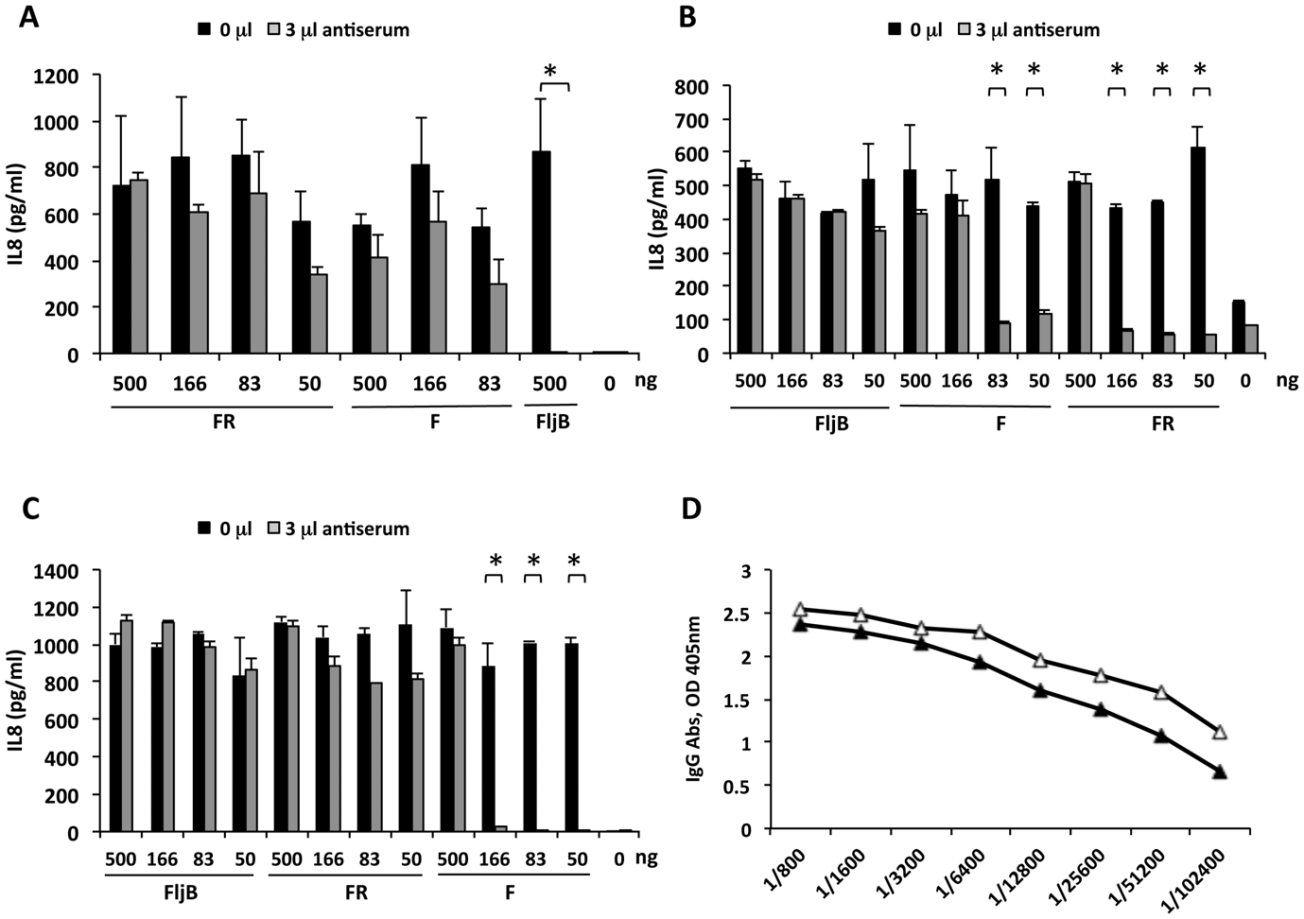
Antibody mediated cross-neutralization of MA and STF flagellins measured by the IL8 secretion by Caco-2 cells. Caco-2 cells were incubated with a premix of 3 µl (grey bars) or 0 µl (black bars) of anti-flagellin hyperimmune serum with MA or STF flagellins. The flagellins (abscissa) indicated were pre-incubated with anti-FljB antiserum (A), anti-FR antiserum (B), and anti-F antiserum (C). (D) Panel showing the IgG titration profile of anti-FljB (▵) and anti-FR (▴).*, statistical significance differences at the p≤0.05 level.

Caco-2 cell cultures were used to evaluate TLR5 activation, as measured by IL8 expression in the presence of pre-mixed flagellin with anti-flagellin antibodies from hyperimmunized mice with a minimum of ELISA titers of 1/100,000 for anti-FljB (**Fig. 5A**) and 1/50,000 for anti-FR and anti-F (**Fig. 5B and 5C**). So, distinct volumes of hyperimmune anti-FljB sera (1 µl, 2 µl and 3 µl) were mixed and incubated with FljB (500 ng, 1 µg/ml) to evaluate the volume required to inhibit TLR5 stimulation *in vitro*. Absence of IL8 expression (full flagellin-TLR5 inhibition) was achieved with 3 µL of antiserum (**Fig. 5A**). This volume of anti-FljB antiserum was then mixed and incubated with a range of concentrations (500, 166, 83 and 50 ng) of F or FR flagellins, and further incubated with Caco-2 cells for 18 h (**Fig. 5A**). Surprisingly, the same amount (500 ng) of either F or FR was not blocked by 3 µl of anti-FljB antiserum. This behavior was maintained even when the concentrations of FR and F were reduced 10 times (50 ng) and 6 times (83 ng) respectively, obtaining similar IL8 expression in the presence or absence of anti-FljB serum (**Fig. 5A**).

Taken together, the results shown in Fig. 5A indicate that hyperimmune anti-STF flagellin sera inhibited STF flagellin but did not block the same or even 10-fold lower amounts of MA flagellins.

In a similar way, we studied the capacity of an anti-FR antiserum to inhibit FR, F and FljB. Interestingly, 500 ng of FR was not inhibited by 3 µl of anti-FR antiserum but was blocked by 166 ng, as well as by lower FR concentrations (**Fig. 5B**). Most probably, FR was active when using 500 ng because there were not enough anti-FR antibodies in 3 µl of antiserum to neutralize it. Thus, in the presence of anti-FR antibodies, F began to lose its capacity to induce IL8 when using 83 ng or lower amounts (**Fig. 5B**). In contrast, FljB maintained its adjuvant capacity even at low amounts (50 ng) as few differences were detected in dose variations in the presence of inhibitory FR antiserum, thus IL8 secretion was similar in the absence of serum (**Fig. 5B**). These results indicate that FljB is active in the presence of anti-FR antibodies that neutralize FR flagellin. In addition, 3 µl of the anti-F serum neutralized 166 ng (or less) of F. Again, anti-F serum did not neutralize FR or FljB at concentrations (166 ng, 83 ng, and 50 ng) at which F was neutralized (**Fig. 5C**). The higher neutralization capacity of the anti-FljB antiserum with respect to the anti-FR (or anti–F) could be due to the specific IgG anti-flagellin antibodies. The anti-FljB antiserum used contained nearly a 2-fold higher IgG titer than the anti-FR antiserum (**Fig. 5D**).

**Figure 6:**
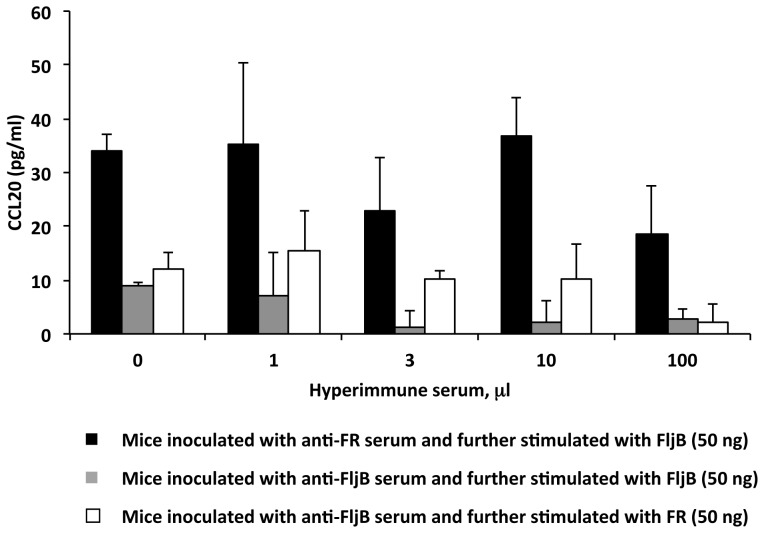
Neutralizing anti-flagellin antisera inoculated by the retro-orbital route do not block heterologous flagellins. CCL20 levels (pg/ml) in sera of mice inoculated by the retro-orbital route with hyperimmune anti-flagellin antiserum 1 h before injection with FljB or FR. A range of amounts of hyperimmune antiserum from 1 to 100 µl were used for retro-orbital inoculation. Mice were stimulated 1 h after serum inoculation with 50 ng of FljB or FR. **Black bars**, mice inoculated with anti-FR antiserum and further stimulated with FljB. **Grey bars**, mice inoculated with anti-FljB antiserum and further stimulated with FljB. **White bars**, mice inoculated with anti-FljB antiserum and further stimulated with FR. Differences in cytokine expression were not statistically significant at the p≤0.05 level.

We further examined *in vivo* cross-neutralization by using different volumes of antiserum (0, 1, 3, 10 and 100 µl) passively transferred to mice by the retro-orbital route and further inoculation of these animals with MA and STF flagellins. Mice receiving 1 µl of the anti-FljB antiserum showed impaired CCL20 induction when stimulated with 50 ng of FljB (**Fig. 6** **, grey bars**), and this amount was completely neutralized when mice were passively transferred with 3–100 µl of anti-FljB serum, although CCL20 expression values between 0 µl and 3 µl were not statistically significant. In contrast, the same amount (50 ng) of FR (**Fig. 6** **, white bars**) was not neutralized by 1–10 µl of anti-FljB antiserum, being able to induce the expression of CCL20 in a similar extent in the groups ranging from 0 µl (only PBS) to 10 µl of the antiserum. Only by using 100 µl of anti-FljB antiserum was 50 ng of FR neutralized, although the difference in CCl20 expression (pg/ml) was not statistically significant with respect to its control (0 µl of anti-FljB). Results shown suggest that FR flagellin may be active when FljB is blocked by neutralizing anti-FljB antibodies.

Thus, mice inoculated with anti-FR antiserum responded to 50 ng of FljB, although the CCL20 expression slightly decreased in mice receiving 100 µl of antisera, and there was not enough concentration of anti-FR antibodies to neutralize FljB (**Fig. 6** **, black bars**).

**Figure 7:**
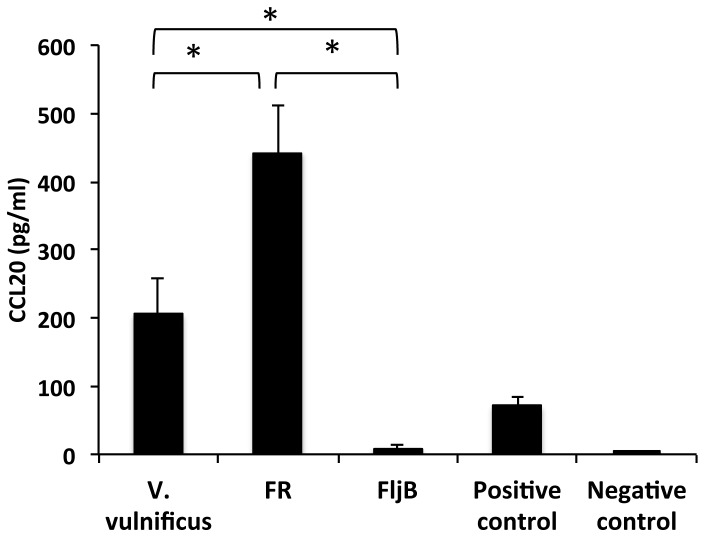
FR flagellin activates the innate immune response in FljB neutralizing mice. CCL20 levels (pg/ml) in sera of FljB-hyperimmunized mice after inoculation with 4.8 µg of FR, FljB or *Vibrio vulnificus* (Vvul or FlaB) flagellins. Positive control, 71 pg of CCL20. Negative control, two naïve-pooled mice. *, differences in the CCL20 amounts induced by FR and Vvul were statistically significant with respect to FljB, and the higher CCL20 expression induced by FR with respect to Vvul was also statistically significant at the p≤0.05 level.

These results were confirmed in FljB-hyperimmunized long-lasting mice. So, groups of these mice received a s.c. injection of FljB, FR or Vvul at a dose of 0.2 mg/Kg of body weight of flagellin (approximately 4.8 µg/mouse), a dosage considered appropriated for vaccination purposes and also to induce radioprotection, via TLR5 activation, from lethal doses between 10 and 13 grays (Gy) [11]. The group of FljB-hyperimmunized mice injected with 4.8 µg of FljB did not respond by inducing CCL20 since the expression of this cytokine was similar to the negative control. This finding suggests that this amount of FljB was fully neutralized by anti-FljB antibodies (**Fig. 7**). However, the groups of FljB-hyperimmunized mice injected with 4.8 µg of either FR or Vvul did show a statistically significant CCL20 induction, this being significantly higher with the FR flagellin with respect to Vvul. These data again demonstrate that FR is active in the presence of neutralizing anti-FljB antibodies. Thus, FR has more capacity to stimulate TLR5 than Vvul or, possibly, the latter is more neutralized by anti-FljB antibodies than FR.

## Discussion

Here we characterized the *in vitro* and *in vivo* functionality of phase 1 and 2 flagellins (FR and F) from MA as adjuvants for the innate and humoral immune response and compared their activity with that of STF (FljB) flagellin.

*In vitro*, F and FR induced similar expression levels of pro-inflammatory cytokines (IL8, CCL2) to FljB in the Caco-2 cell line. However, *in vivo*, the humoral immune response induced by MA flagellins delivered by the systemic route (s.c.) differed slightly. While F4DUD evoked very similar specific anti-DUD antibody levels to FljB4DUD, FR4DUD induced titers almost 2 times lower. Thus, F4DUD delivered by the i.n. route generated half the titer of anti-DUD antibodies than F4DUD by the s.c. route. This observation is in concordance with data from previous studies that report a more potent pro-inflammatory effect of flagellins when delivered by the systemic route. However, the fact that FR4DUD induced lower anti-DUD titer values than F4DUD cannot be explained by its inefficiency to activate TLR5. Our data showed that FR induced the expression of pro-inflammatory cytokines (IL8, CCL2) with similar strength as F and FljB. Thus all the flagellins examined here have the capacity to activate TLR5 and, therefore, to support functions such as dendritic cell maturation [58]. Recently, it has been demonstrated in a mouse model that flagellin promotes adaptive immunity without the robust activation of TLR5 and signal transduction through MyD88 [58]. Therefore, in addition to differences in the primary sequence of FR4DUD with respect to F4DUD and FljB4DUD flagellins, other mechanisms may act in response to flagellin and could be relevant for the development of a specific mediated Ig response against DUD. More studies with other model antigens fused to MA flagellins are required to clarify this issue. On the other hand, anti-F flagellin antibodies have lower cross-reactivity with STF than anti- FR ones. Together, these data suggest that MA-F flagellin is a better adjuvant than MA-FR when using in sequential combination with STF.

Regarding cross-neutralization, *in vitro* and *in vivo* assays showed that serum from FljB- hyperimmunized mice inhibited FljB but not FR or F, even at 6- to 10-fold lower amounts of MA flagellins (**Fig. 5** **,** **6** **and** **7**). A similar behavior was observed for the serum from FR-hyperimmunized mice; the anti-FR antibodies inhibited FR and F at low amounts, but not FljB.

Total IgG quantification data from anti-FljB serum yielded 7 mg/ml whereas anti-FR produced 4 mg/ml. These values may explain why anti-FljB antiserum neutralized more FljB than that achieved by the anti-FR antiserum for FR. Despite the variations observed in IgG titers against each flagellin, the most relevant data are that a high concentration of anti-FljB antibodies did not inhibit FR and F at concentrations used in *in vitro* assays, and antibodies against FR and F did not neutralize FljB at low concentrations.

Importantly, these results were confirmed *in viv*o since at dosages similar to those used for either radioprotection or vaccination (4.8 µg, 0.2 mg/kg), the MA FR was fully functional against FljB in hyperimmunized mice, whereas FljB was again blocked. Furthermore, at that dose, Vvul was partially neutralized since the level of pro-inflammatory CCL20 was half that obtained with FR. This observation suggests that it would be advantageous to use FR instead of Vvul as an adjuvant for FljB- hyperimmunized mice.

To our knowledge, this is the first study to demonstrate the contribution of anti-flagellin antibodies to the neutralization of flagellins of another bacterial species. Previous research [59] showed that a mutated flagellin from *P. aeruginosa*, which carried arginine instead of alanine at position 90 (FliCR90A), was unable to activate TLR5 but still induced protective antibodies against *P. aeruginosa*, although with weak cross-neutralization of its FliC. This mutation strategy is appropriate for particular pathogenic bacteria but not for activating TLR5. In addition, in that study [59], the immunizations were carried out using DNA vaccines and not protein. These distinct antigen delivery systems may imply differences in anti-flagellin antibody responses.

Our data show that flagellin neutralization is mediated by antibodies not only against the domain that binds to TLR5 [60] but also to other amino acids that are close or relatively close to this domain and that could hamper the steric approach of flagellin to the TLR5 and the consequent activation of this receptor. The latter is bolstered because flagellins carrying small proteins fused to their N-terminal domain decrease IL8 release by 50% as a result of reduced TLR5 activation with respect to wild-type flagellins [[9] and our own results, data not shown]. Thus, the amount of specific antibodies against domain D1, and specifically to amino acid positions 89–96, would not be dominant with respect to other parts of the molecule, for example against the hypervariable region, meaning that the 89–96 sequence is not an immunodominant epitope in B- and T-cell immune response against flagellin. This notion is supported by the observation that antibodies from hyperimmune antisera against FljB, F or FR flagellins neutralize themselves, but not each other. However, this domain is essential for TLR5 activation, as demonstrated by the generation of non-functional mutated or deleted flagellins at these amino acid positions [4], [43]. In spite of this, a monoclonal antibody against this region blocks TLR5 activation [60].

A recent study reported crystallographic data about the complex interactions between flagellin and TLR5 from *Danio rerio*, in which carboxy- and amino- terminal parts of D1 are involved in conforming primary interfaces B and A, respectively. These primary interfaces help to form a 1∶1 flagellin-TLR5 dimer and further dimerization [61]. It appears that differences between primary amino acid sequences of the STF and MA flagellins examined in the present study are not large enough to show discrepancies in TLR5 activation by means of measuring the expression of the pro-inflammatory cytokine IL8 and IgG antibody production against DUD antigen model induced by FljB4DUD and F4DUD flagellins.

Our findings demonstrate the functional capacity of MA flagellins (FR or F) as independent adjuvants and the advantage of their administration in a sequential combination with flagellins derived from STF (FljB or FliC) to trigger TLR5 activation. So, this characteristic is especially relevant in individuals previously vaccinated with STF flagellins or in those previously infected with *Salmonella*, because MA flagellins avoid the neutralization of anti-flagellin antibodies from STF generated after exposure by the various routes (intra-nasal, intra-peritoneal, intra-muscular). However, more studies are required to address whether other bacterial flagellin molecules (e. g. *Vibrio spp.*) are neutralized by anti-FljB or anti-F/FR antibodies, thereby increasing the range of further applications.

In individuals with varying degrees of pre-existing antibody expression against STF flagellins, MA flagellins may induce TLR5 activation through much smaller amounts than STF. Regarding safety, this point is relevant in order to avoid the administration of very large or crescent amounts of STF flagellins to be functional, which may produce shock as a result of excessive activation of pro-inflammatory genes [62]. On the other hand, in non-STF exposed individuals, MA-F flagellin would be more appropriate than MA-FR when using as first adjuvant because gives less cross-reaction with STF delivered as second adjuvant.

Our data may be useful for future applications of flagellin-based therapeutic strategies, such as vaccines, antitumoral therapies, treatments against acute organ failure and acute radiation syndrome, and also to improve the outcome of bone marrow transplants.
